# Genetic Analysis of Variation in Human Meiotic Recombination

**DOI:** 10.1371/journal.pgen.1000648

**Published:** 2009-09-18

**Authors:** Reshmi Chowdhury, Philippe R. J. Bois, Eleanor Feingold, Stephanie L. Sherman, Vivian G. Cheung

**Affiliations:** 1 Department of Pediatrics, University of Pennsylvania, Philadelphia, Pennsylvania, United States of America; 2 The Scripps Research Institute, Genome Plasticity Laboratory, La Jolla, California, United States of America; 3 Departments of Human Genetics and Biostatistics, Graduate School of Public Health, University of Pittsburgh, Pittsburgh, Pennsylvania, United States of America; 4 Department of Human Genetics, Emory University School of Medicine, Atlanta, Georgia, United States of America; 5 Department of Genetics, University of Pennsylvania, Philadelphia, Pennsylvania, United States of America; 6 Howard Hughes Medical Institute, University of Pennsylvania, Philadelphia, Pennsylvania, United States of America; The University of North Carolina at Chapel Hill, United States of America

## Abstract

The number of recombination events per meiosis varies extensively among individuals. This recombination phenotype differs between female and male, and also among individuals of each gender. In this study, we used high-density SNP genotypes of over 2,300 individuals and their offspring in two datasets to characterize recombination landscape and to map the genetic variants that contribute to variation in recombination phenotypes. We found six genetic loci that are associated with recombination phenotypes. Two of these (*RNF212* and an inversion on chromosome 17q21.31) were previously reported in the Icelandic population, and this is the first replication in any other population. Of the four newly identified loci (*KIAA1462*, *PDZK1*, *UGCG*, *NUB1*), results from expression studies provide support for their roles in meiosis. Each of the variants that we identified explains only a small fraction of the individual variation in recombination. Notably, we found different sequence variants associated with female and male recombination phenotypes, suggesting that they are regulated by different genes. Characterization of genetic variants that influence natural variation in meiotic recombination will lead to a better understanding of normal meiotic events as well as of non-disjunction, the primary cause of pregnancy loss.

## Author Summary

Meiotic recombination is essential for the formation of human gametes and is a key process that generates genetic diversity. Given its importance, we would expect the number and location of exchanges to be tightly regulated. However, studies show significant gender and inter-individual variation in genome-wide recombination rates. The genetic basis for this variation is poorly understood. In this study, we used genotypes from high-density single nucleotide polymorphism (SNP) markers of 2,315 individuals and their children from two Caucasian samples in a genome-wide association study to identify genetic variants that influence the number of meiotic recombination events per gamete. We found three loci that influence female recombination and three different loci that influence male recombination. Our results suggest that gender differences in recombination result from differences in the genetic regulation of female and male meiosis. Also, each identified locus only explains a small proportion of variance; together, each set of loci explains about 10% of the variation in the gender-specific recombination phenotype. This suggests a mechanism for variability in recombination that is essential for genetic diversity while maintaining the number of recombinations within a range to ensure proper chromosome segregation.

## Introduction

Meiotic recombination is essential for cell division and is a key process that generates genetic diversity. It provides daughter cells with allelic compositions that differ from those of their parents. However, despite its important role, recombination frequency differs significantly between females and males, and also among individuals within each gender [1],[2],[3]. Gender differences in recombination rates are also seen in other organisms, such as mice [4],[5].

Errors in meiotic recombination lead to chromosomal abnormalities including nondisjunction; thus cellular processes must ensure proper meiotic recombinations [6],[7]. Proteins such as those involved in double-strand DNA breaks are known to be involved in recombination; however regulatory processes and mechanisms by which DNA breaks in meioses resolve into crossovers remain unknown [8],[9],[10]. The variation in recombination rates offers an opportunity to identify regulators of this key cellular process. By treating recombination rate as a quantitative trait, we can screen the genome for DNA variants that influence this process without knowing a priori the regulatory mechanisms.

The genetic basis of individual differences in human meiotic recombination is poorly understood. An inversion on chromosome 17q21.31 [11] and sequence variants in *RNF212* [12] are the only known genetic determinants. In this study, we used genotypes from high-density single nucleotide polymorphism (SNP) markers of 2,315 individuals and their children from two Caucasian samples to characterize meiotic recombinations. We treated the number of recombinations per meiotic event as a quantitative phenotype (from hereon we will refer to this as recombination phenotype) and carried out a genome-wide association study (GWAS). From the >137,000 female recombination events and >87,000 male recombinations events in the two datasets, we found significant individual variation in the numbers and locations of recombination events. We identified six genetic loci that show allelic association with female and male recombination phenotypes. Among them are the sequence variants on chromosome 17q21.31 and those in *RNF212* that were previously reported to be associated with recombination phenotypes in females and males, respectively. The remaining four loci were not known to contribute to individual variation in genome-wide recombination rates. These results provide new information to study the regulation of meiotic recombination.

## Results

### Recombination phenotype

**Figure 1:**
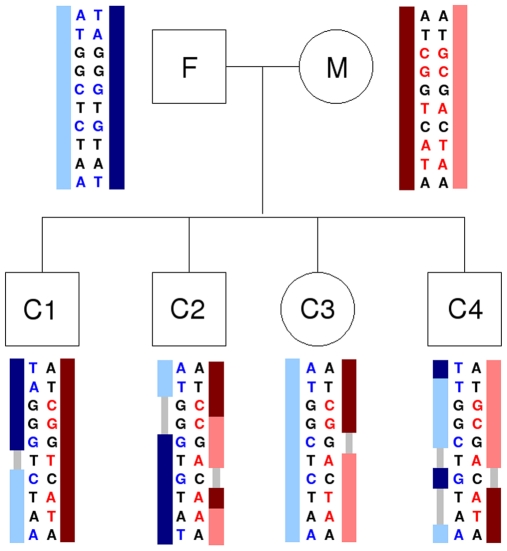
Identification of recombination events. Genotypes at 10 consecutive SNPs for two parents and their four children in this pedigree are provided. Informative makers are marked in red and blue for the mother and father, respectively. Recombination events are shown by color switches (for example from dark to light red), or, if regions are large, they are shown in gray.

**Figure 2:**
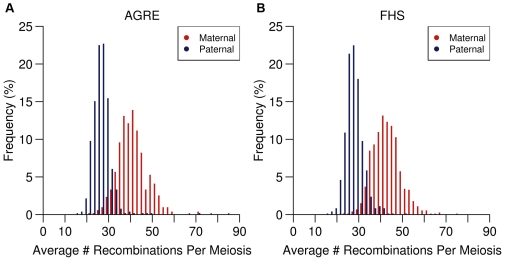
Distribution of recombination phenotypes. Histograms showing the distributions of maternal (red) and paternal (blue) recombinations in the AGRE (A) and FHS (B) samples.

We used genotypes from the Autism Genetic Research Exchange (AGRE) [13] and Framingham Heart Study (FHS) [14] collections to determine recombination phenotypes. For our analysis, we used the genotype data from members of two-generation families that have two or more children to infer recombination phenotypes of the parents in these families. The 511 AGRE families have an average of 2.26 children (median = 2; range: 2 to 7) and provided data for 1,155 female and 1,155 male meioses. Using ∼400,000 SNP genotypes of the parents and children in these families, we inferred the recombination phenotypes of 511 mothers and 511 fathers. Briefly, we used the genotypes of the parents to identify informative markers. Then, using these markers, we compared the genotypes of the children to determine the alleles that they had inherited identical-by-descent from the mothers and fathers. Between two sibs, a switch from sharing the same maternal allele to the different maternal allele was scored as a maternal recombination event; and same for the sharing of paternal alleles (see Materials and Methods, Figure 1). From analysis of these AGRE families, we identified 47,573 female recombination events and 30,578 male recombination events over the 22 autosomes (see Table S1). The average number of maternal recombinations per meiosis was 41.1 (95% CI: 39.9–42.4), and the average number of paternal recombinations per meiosis was 26.4 (95% CI: 25.7–27.2). This is consistent with previous human studies which show that there are more recombination events in female meiosis than in male meiosis. The female∶male ratio in the AGRE dataset is 1.6, which is very similar to those in previous studies of CEPH (1.6) [1],[2], Icelandic families (1.65) [15] and Hutterites (1.5) [3]. The distributions of recombination events for females and males in the AGRE collection are shown in Figure 2A.

For the second population, we analyzed genotypes for ∼500,000 SNP markers from members of 784 two-generation families from the FHS. This dataset provided us with recombination phenotypes for 654 mothers and 639 fathers, with an average of 2.86 children per individual (median = 3; range: 2 to 9). We observed 90,264 female and 57,054 male recombinations (Table S1). The average number of maternal recombinations per meiosis was 42.8 (95% CI: 42.4–43.3), and the average paternal recombinations per meiosis was 27.6 (95% CI: 27.3–27.9). The female∶male ratio was also 1.6. The distributions of female and male recombination events per meiosis for individuals in the FHS collection are shown in Figure 2B.

We compared the recombination phenotypes in the AGRE and FHS collections (and also with those from previous studies) and found highly similar patterns. Previous literature reports mean maternal genome-wide recombination ranging from 38.4 to 47.2, and mean paternal genome-wide recombination ranging from 25.9 to 27.3 [2],[3],[12],[15]. The mean recombination phenotypes for AGRE and FHS fall within, or very close to, the ranges in the published data. We also compared the resolution of our ability to map crossovers with that of Coop et al. [3]. From our two samples we mapped 40,942 (∼18%) recombinations to regions that are <30 kb in size; similarly they identified 4,854 (∼20%) recombinations to regions <30 kb in size. Because of our larger sample size, we identified more recombinations but the resolutions in the two studies are comparable.

### Recombination jungles

Recombination events are not distributed evenly across the human genome [15]. We refer to genomic regions with higher recombination counts are referred to as “recombination jungles” [2],[15] (rather than hotspots, which are only hundreds of base pairs in size). To identify the location and size of recombination jungles in the AGRE and FHS samples, we sorted and plotted all recombination events by base pair position. The peaks in the derivative function of curves fitted to the recombination events were identified as recombination jungles (see Materials and Methods and Figure S1). Previously, to identify recombination jungles, we divided the genome into equal-size bins where bin sizes were picked arbitrarily [2]. The approach we used here allows us to identify jungles based on distribution of SNPs and recombination activities in different genomic regions, thus the results should better reflect the actual recombination activities.

**Figure 3:**
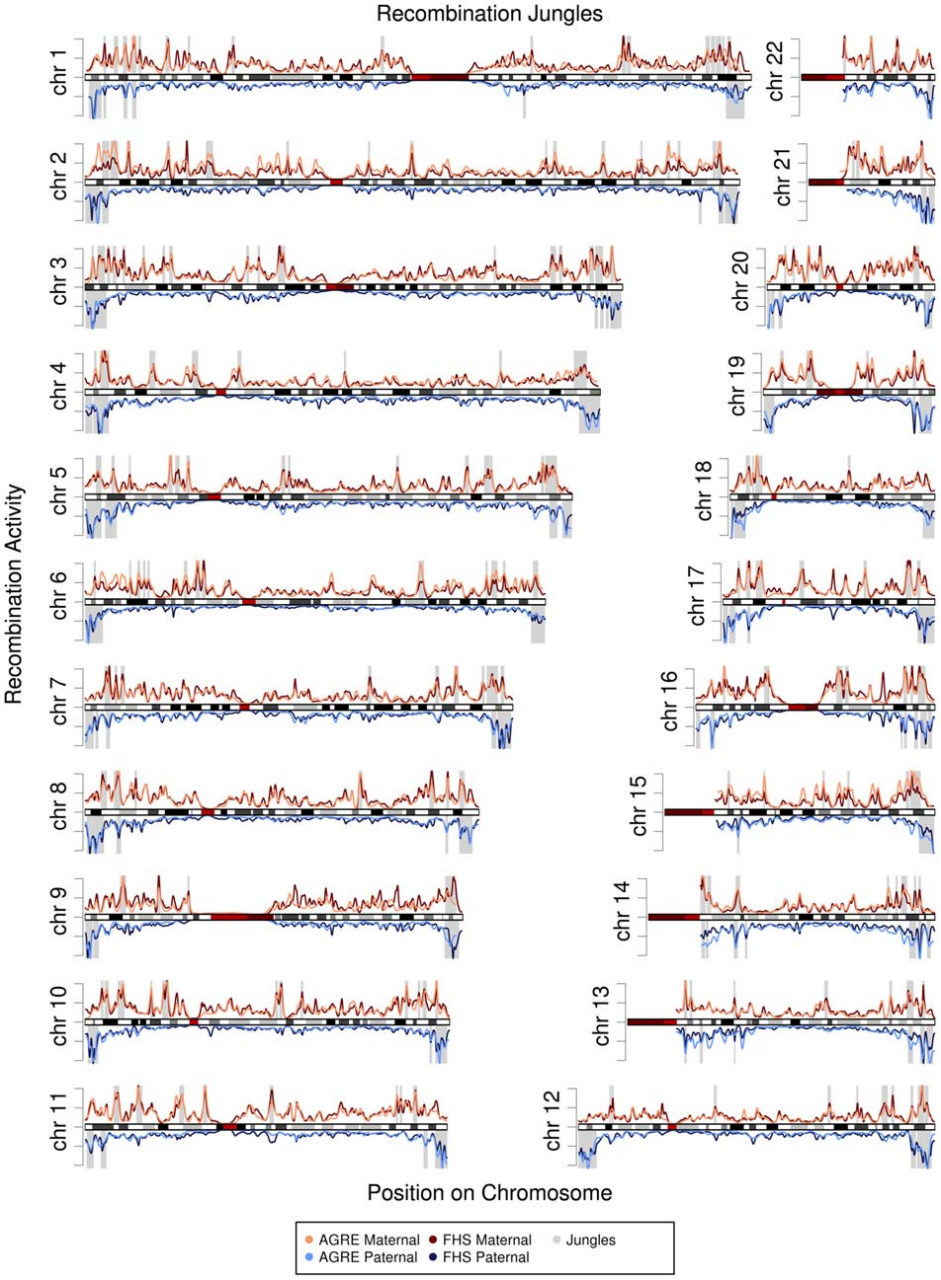
Recombination jungles across the human genome. The curves in each graph represent the intensity of recombination activity across the chromosome. Regions with the most recombination activity (2 standard deviations above the mean) are shaded in gray and are considered “recombination jungles.” Recombination events for females (top) and males (bottom) are shown for the AGRE (pink and light blue) and FHS (red and dark blue) samples. Chromosome ideograms show centromeres in red and patterns of Giemsa staining in different shades of grey.

Using this approach, we identified 125 maternal recombination jungles and 69 paternal recombination jungles in the AGRE population. The average size of the maternal jungles was 2.1 Mb (range: 0.8 to 6.0), and that of the paternal jungles was 3.7 Mb (range: 1.1 to 11.1). In the FHS population, we identified 183 maternal recombination jungles, averaging 1.5 Mb (range: 0.5 to 4.9), and 86 paternal jungles, averaging 2.7 Mb (range: 0.5 to 8.6). The positions and sizes of recombination jungles throughout the genome were very similar for individuals from the AGRE and FHS collections (Figure 3).

Recombinations tend to occur in the telomeric parts of chromosomes. Most of the paternal recombination jungles were found at the telomeric ends of each chromosome (Figure 3), while the maternal recombination jungles were found at the ends of the chromosomes but not always at the most telomeric parts (Figure 3). Seventy percent of male recombination jungles are located in the 5% most telomeric parts of each chromosome, while 18% of the female recombination jungles are found in the same regions. Previously, in the CEPH data, we also found that recombination jungles were found at the ends of chromosomes [2]. However, data from AGRE and FHS have provided a finer scale map of the recombination activities across the genome.

### Individual differences in recombination phenotype

We and others have shown that there are extensive individual differences in recombination phenotypes in both females [1],[2] and males [2],[3]. Since the AGRE and FHS samples are larger than those used for previous studies, we use the AGRE and FHS samples to assess individual variation in recombination phenotypes. For individuals with only two offspring, we are not able to identify which recombinations occur in which offspring (see Materials and Methods), but for individuals with three or more offspring we can count recombinations individually in each offspring and thus we have repeated measures for each parent. Using parents with three or more offspring (119 AGRE mothers, 119 AGRE fathers, 374 FHS mothers, and 356 FHS fathers), we conducted an analysis of variance to compare the variability of recombination in different meiotic events (offspring) within an individual to the variability between individuals, and found highly significant individual differences in mean recombination frequency among men (P_AGRE_ = 5.14×10^−12^, P_FHS_ = 2.83×10^−49^) and women (P_AGRE_ = 1.09×10^−16^, P_FHS_ = 8.97×10^−18^) in both samples.

### Genome-wide association analysis

To identify the DNA variants that influence individual variation in recombination phenotype, we carried out genome-wide association analysis. Since the distributions of female and male recombination phenotypes are different, for all the analyses, we studied female and male recombination phenotypes separately.

**Figure 4:**
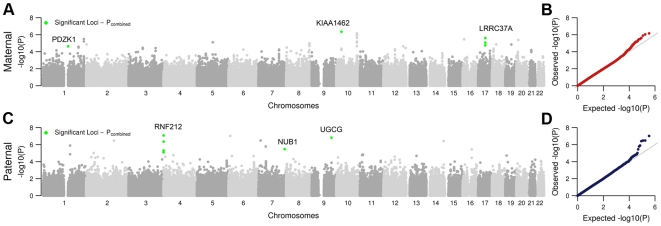
Results of genome-wide association studies of female and male recombination phenotypes. Results of analysis of the AGRE samples for females (A) and males (C) are shown. Combined P-values for the significant loci are plotted in green. Quantile–Quantile plots with observed P-values plotted as a function of expected P-values for female (B) and male (D) recombination phenotypes are shown.

First, we analyzed data from 511 females and 511 males from the AGRE samples. We treated the recombination phenotypes as quantitative traits. For genotypes, we used ∼350,000 SNP genotypes that passed a quality filter. Then, we tested for association of the recombination phenotypes with SNP alleles using an additive model. A plot of the GWA results and a QQ-Plot are shown in Figure 4. Among the significant SNPs are ones in *RNF212*, which was reported by Kong et al to be associated with recombination rate in the Icelandic population [12]. The most significant SNP (rs11939380) within *RNF212* has a P-value of 0.0009 in the paternal AGRE sample.

#### Replication of significant markers in FHS

To replicate the findings from the AGRE samples, we followed up approximately 0.5% of the markers (1,633 maternal markers and 1,766 paternal markers) with further analysis in the 654 females and 639 males in the FHS sample. Many of the markers were found in blocks of linkage disequilibrium and are highly correlated.

Table 1 lists the male and female markers that were most significantly associated with female and male recombination (P-values between 10^−8^ and 10^−5^). For females, the list includes the inversion on chromosome 17q21.31 that was previously reported to be associated with recombination rate in the Icelandic population [11]. In addition, SNP alleles on chromosome 1q21.2 and 10p11.23 were also found to be significantly associated with recombination phenotypes. For the paternal recombinations, six markers at 3 different loci (4p16.3, 7q36.1, 9q31.1) are listed; this includes the previously reported SNPs in *RNF212* on chromosome 4.

**Table 1:** Most significant results from genome-wide association analysis of recombination phenotypes.

MATERNAL												
				AGRE			FHS					
RSID	Chr	Loc	SNP*	N	P	β	N	P	β	Combined P	Nearest Gene	Distance (BP)
rs1797052	1	1q21.1	CT	511	0.00452	2.424	653	0.00083	2.515	2.34×10^−5^	*PDZK1*	−50
rs2505089	10	10p11.23	CA	511	0.00006	2.546	650	0.00088	1.686	4.42×10^−7^	KIAA1462	−1000
rs4640231	17	17q21.31	GC	501	0.00418	1.668	645	0.00066	1.441	1.77×10^−5^	*CRHR1*	0
rs2732705	17	17q21.31	TG	500	0.00222	1.927	650	0.00056	1.506	8.42×10^−6^	*LRRC37A*	−20000
rs2668622	17	17q21.31	GT	498	0.00173	2.014	639	0.00019	1.680	2.45×10^−6^	*LRRC37A*	−20000

PATERNAL												
				AGRE			FHS					
RSID	Chr	Loc	SNP*	N	P	β	N	P	β	Combined P	Nearest Gene	Distance (BP)
rs6827357	4	4p16.3	AC	510	0.00131	1.054	633	0.00089	0.908	7.96×10^−6^	*RNF212*	6000
rs2014318	4	4p16.3	AG	509	0.00103	1.084	613	0.00066	0.907	4.83×10^−6^	*RNF212*	0
rs11939380	4	4p16.3	TC	510	0.00089	0.963	638	0.00001	1.011	8.25×10^−8^	*RNF212*	0
rs4045481	4	4p16.3	GA	510	0.00133	0.922	627	0.00004	0.967	4.46×10^−7^	*RNF212*	0
rs11764733	7	7q36.1	CG	510	0.00387	0.818	638	0.00012	0.885	3.39×10^−6^	*NUB1*	1100
rs7863596	9	9q31.3	AG	498	0.00013	2.213	639	0.00013	1.849	1.51×10^−7^	*UGCG*	−30000

*: The underlined allele is associated with higher recombination rate.

#### Genetic loci associated with female recombination phenotypes

**Figure 5:**
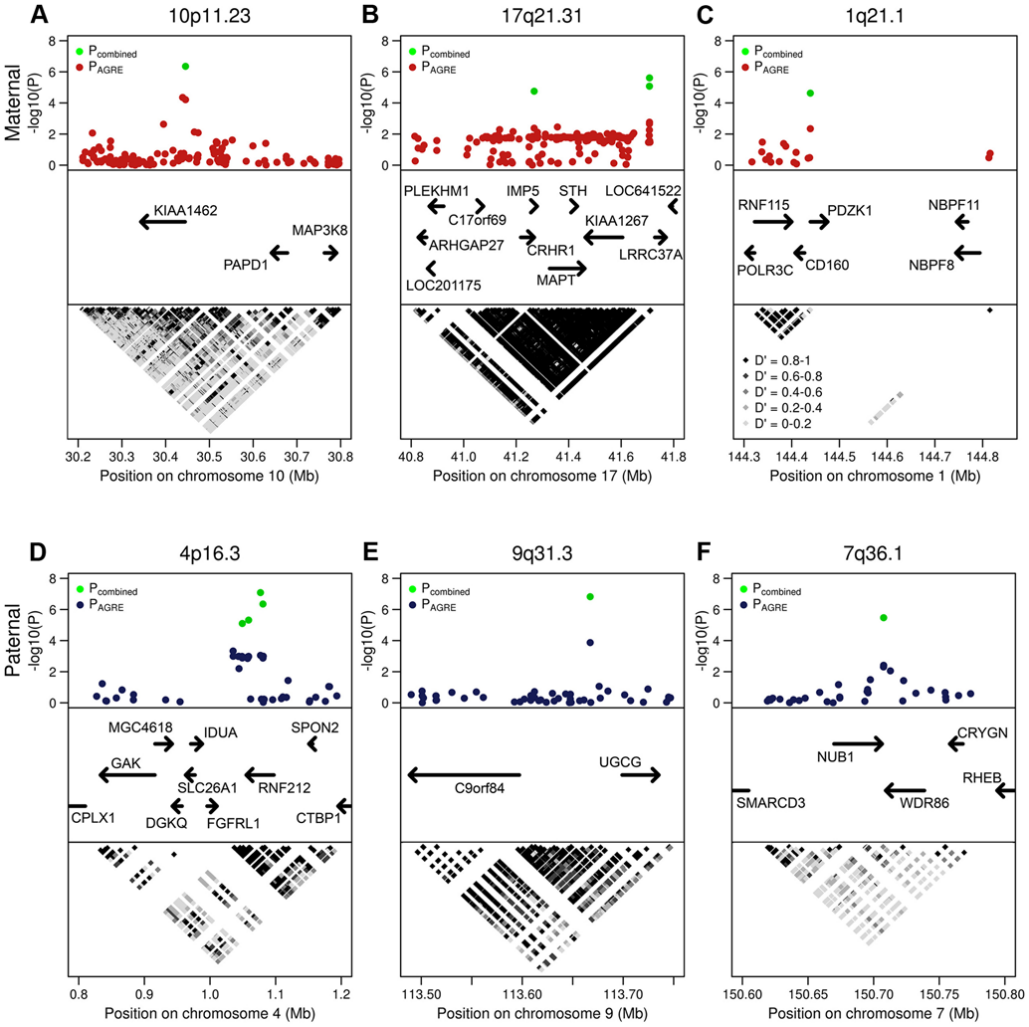
Genomic regions that include the 6 most significant loci associated with recombination phenotypes. (A–C) Maternal association results for SNPs from analysis of the AGRE samples (red) and combined AGRE and FHS samples (green) plotted for loci on chromosomes 10p.11.23 (A), 17q21.31 (B), and 1q21.1 (C). (D–F) Paternal association results for SNPs from analysis of the AGRE samples (blue) and combined AGRE and FHS samples (green) plotted for loci on chromosomes 4p16.3 (D), 9q31.3 (E), and 7q36.1 (F). The top panel of each figure highlights the most significant SNPs in the region. The center panel of each figure shows genes in each region, and direction of transcription. The bottom panel of each figure shows LD patterns at each locus, where black corresponds to stronger LD and light gray corresponds to weaker LD as shown in (C).

The strongest maternal association signal was observed on chromosome 10p11.23 at marker rs2505089, the P-value for the combined AGRE and FHS samples was 4.4×10^−7^ (Table 1; Figure 5A). This SNP is about 1 kb downstream of *KIAA1462*, a poorly characterized gene. We queried gene expression data from the GNF expression atlas [16] and those in NCBI GEO [17] (GDS1266, GDS2223, GDS3254, GDS665) and found that *Kiaa1462* is highly expressed in ovaries of newborn mice, and in oocytes in meiotic prophase I.

The second most significant result was found on chromosome 17q21.31 (Figure 5B) at SNP rs2668622 (P_combined_ = 2.4×10^−6^). There were two additional significant markers at this locus: rs4640231 (P_combined_ = 1.7×10^−5^) and rs2732705 (P_combined_ = 8.4×10^−6^). All three markers are highly correlated, with pairwise R^2^>0.8, and reside within a 900 kb region of strong LD. SNPs rs2668622 and rs2732705 are both approximately 20 kb upstream from gene *LRRC37A*, while SNP rs4640231 resides within an intron of gene *CRHR1*. This locus contains several additional genes, including *IMP5*, *MAPT*, *STH*, and *KIAA1267* (Figure 5B). Stefansson and colleagues previously reported that a common inversion, H2, in this region is associated with recombination rates [11]. The frequency of the H2 haplotype is 18% and 23% in the AGRE and FHS samples, respectively, which is very close to the 20% estimated by Stefansson and colleagues. In our two samples, the rs1800547 G-bearing H2 haplotype is also associated (P_combined_ = 8.9×10^−6^) with higher recombination frequency.

The third maternal association signal was on chromosome 1q21.1 (Figure 5C) at SNP rs1797052 (P_combined_ = 2.3×10^−5^). SNP rs1797052 is approximately 50 bp upstream of gene *PDZK1* which encodes a scaffold protein that regulates ion transport and second messenger cascade in epithelial cells [18]. It is previously not known to play a role in meiotic recombination. Gene expression data (GDS3254 and GDS2203) in the NCBI GEO database [17] show that *Pdzk1* is expressed in ovaries of newborn mice where the oocytes are in prophase I.

To evaluate the collective impact of the three significant loci on the maternal genome-wide recombination phenotype, we built a model using stepwise linear regression. Markers rs1797052, rs2505089, and rs4640231 were used as predictor variables. The three loci explain 7.3% (P = 3.56×10^−8^) of variation in recombination phenotype in the AGRE sample, 4.75% (P = 1.14×10^−6^) of the variation in FHS, and 5.9% (P = 1.77×10^−14^) variation in combined AGRE and FHS samples.

#### Genetic loci associated with male recombination phenotypes

The strongest association signal for paternal recombination phenotype was observed on chromosome 4p16.3 at SNP rs11939380 (P_combined_ = 8.2×10^−8^) (Table 1, Figure 5D). Three additional significant markers were identified in this region: rs6827357 (P_combined_ = 7.9×10^−6^), rs2014318 (P_combined_ = 4.8×10^−6^), and rs4045481 (P_combined_ = 4.4×10^−7^). All four markers reside within a strong LD block that includes RNF212, a gene that was previously reported to be associated with meiotic recombination by Kong and colleagues [12]. The most significant marker in the Kong study (rs3796619) was part of a haplotype found to decrease genome-wide male recombination by 70.7 cM. In our analysis, the same haplotype decreases male recombination rates by ∼101 cM in the AGRE and ∼95 cM in the FHS.

**Figure 6:**
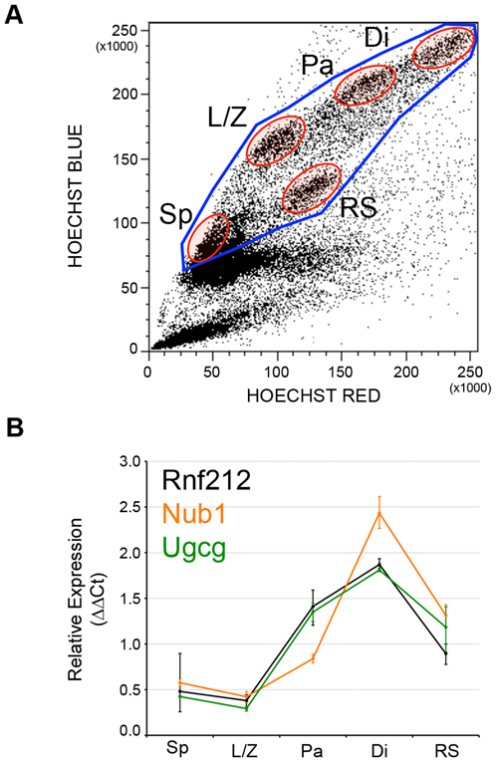
Expression analysis of genes associated with male recombination phenotypes. (A) Mouse male meiotic cells were purified by FACS from testes. A typical profile is shown with the purified cell fractions circled in red. All meiotic cells were also purified and used as reference sample (circled in blue). (B) Expression patterns for genes associated with male recombination phenotypes. All genes display a strong induction from the onset of meiosis. Relative expression (ΔΔCt) was calculated using mouse β-actin as the endogenous control and the entire meiotic cells as the reference. Error bars indicate ± s.e.m. Sp: Spermatogonia, L/Z: Leptotene/Zygotene, Pa: Pachytene, Di: Diplotene, SS/RS: Secondary spermatocytes and round spermatids.

The second strongest paternal association signal was found on chromosome 9q31.3 at marker rs7863596 (P_combined_ = 1.5×10^−7^) (Figure 5E). This marker is located about 30 kb upstream of *UGCG* which catalyzes the first glycosylation step in glycosphingolipid biosynthesis. This gene is not known to play a role in meiosis. Using flow cytometry [19],[20] (Figure 6A) and reverse transcription (RT) - PCR, we found that *Ugcg* is induced during meiosis with the highest expression at the diplotene stage which corresponds to chromosome chiasmata resolution (Figure 6B). We also observed that the expression profiles for *Ugcg* and *Rnf212* are highly similar (Figure 6B).

The third paternal association signal was on chromosome 7q36.1 at marker rs11764733 (P_combined_ = 3.3×10^−6^) (Figure 5F). This marker is approximately 1 kb downstream of *NUB1*, which according to our expression analysis, is also induced as meiosis progresses with a peak at the diplotene stage (Figure 6B).

We used markers rs11939380, rs11764733, and rs7863596 in a stepwise linear regression model to quantify the combined impact of the three loci on individual variation in genome-wide paternal recombination phenotype. The three loci account for 6.8% of variation in AGRE (P = 1.22×10^−7^), 5.0% of variation in FHS (P = 1.28×10^−6^), and 5.4% of variation in the combined AGRE and FHS (P = 4.62×10^−13^).

#### Analysis combining female and male recombination phenotypes

In the above analysis, we studied the female and male recombination phenotypes separately; we also carried out GWAS using the phenotypes in a combined analysis. We first normalized the means of the female and male recombination phenotypes to adjust for the gender differences, and carried out GWAS as above. Using the same thresholds, we did not find any markers with significant allelic association with the combined recombination phenotypes.

## Discussion

We used a sample of 1,295 two-generation families with multiple offspring to study the recombination landscape and the genetic basis of meiotic recombination. Our analysis showed that the locations of the recombination events differ across the genome; most of the crossovers occur at the ends of chromosomes. In particular, 70% of male recombination jungles are located in the 5% most telomeric parts of chromosomes. This pattern is observed in samples from the AGRE and FHS collections and also in previous studies of CEPH and Icelandic populations. We also found extensive individual variation in the number of recombination events per meiosis in both females and males. To determine the genetic basis of this variation, we carried out genome-wide association analysis. We treated the number of recombination events as quantitative traits to map the genetic variants that influence recombination phenotype. We found three loci that influence the female recombination phenotype, and three loci that influence the male recombination phenotype.

Our GWAS analysis replicated the previous findings that an inversion on chromosome 17q21.31 is associated with higher female recombination rates, and that variants in *RNF212* influence recombination rates in males. The previous work showed association between haplotypes in *RNF212* and male and female recombination, with opposite effects in the two sexes [12]. Specifically, they found SNP rs1670533 associated with female recombination and rs3796619 associated with male recombination. Neither of these SNPs was in the set we examined, but the linkage disequilibrium in this region is very high, and our dataset does contain SNPs that are perfect surrogates (*r* ^2^ = 1.0) in HapMap for each of these. Our surrogates for the Icelandic male SNP include rs4045481 and rs11939380, both of which had P-values on the order of 10^−6^ for male recombination in our combined dataset. Our surrogates for the Icelandic female SNP are rs6827357 and rs20114318. Both of these showed P-values on the order of 0.01 for females in our combined dataset. As in the Icelandic population, we observed opposite effects on male and female recombination at these SNPs and throughout the haplotype block. While the P-values for female recombination in our dataset fall far short of genome-wide significance, they do show a weak association and are quite consistent with the Icelandic results. It is particularly notable that this is the first replication of the curious opposite effects on male and female recombination previously observed.

In addition to these known loci, we uncovered additional polymorphic regions that are associated with recombination phenotypes. In females, we found variants on chromosome 10 near a poorly characterized gene, *KIAA1462*, and those on chromosome 1 near *PDZK1* to be associated with recombination rates. These variants along with those on chromosome 17q explain approximately 6% of the total variation in female recombination phenotype in the AGRE and FHS samples. In males, we identified variants on chromosome 9 near *UGCG* and on chromosome 7 near *NUB1* to be associated with recombination phenotype. In the mouse, the expression of *Ugcg* and *Nub1* in prophase I further supports their potential roles during meiosis. The variants in *RNF212* and those on chromosomes 7 and 9 explain about 5.4% of variation in male recombination.

Results from our genetic mapping study enhanced our understanding of meiotic recombination. It appears that gender differences in recombination rates and pattern result from differences in the regulation of female and male meiosis. Our genetic mapping results showed that DNA variants in different genes are associated with female and male recombination phenotypes; we did not find any variants that are significantly associated with both female and male recombination phenotypes. Second, we identified multiple unlinked SNPs that are associated with recombination phenotypes suggesting that multiple polymorphic regulators influence these phenotypes. This likely provides a mechanism for variability in recombination which is essential for genetic diversity while maintaining the number of recombination events within a range that ensures proper disjunction. Each of the variants that we identified explains only a small fraction of the individual variation in recombination. Together the three loci that contribute to female recombination explain less than 10% of the variation, and the same for male recombination. Unlike most essential cellular processes, recombination events must differ between individuals to maintain genetic diversity. However, the system cannot be so flexible that it fails to ensure proper segregation of chromosomes. Having many regulatory steps achieves the goal of allowing some range of events to occur while ensuring that the number of recombination events does not deviate too much to cause improper chromosome segregation or non-disjunction. Although, we have identified six variants that influence recombination events, we expect other variants still need to be identified. Characterization of genetic variants that influence natural variation in meiotic recombination will allow a better understanding of normal meiotic events as well as non-disjunctions which lead to chromosomal abnormalities, the primary cause of miscarriages.

## Materials and Methods

### Genotype data/population information

We obtained SNP genotypes from samples in two collections: Autism Genetic Research Exchange (AGRE) [13] and Framingham Heart Study (FHS) [14]. For our analysis, we used the genotype data from members of two-generation families that have two or more children to infer recombination phenotypes of the parents in these families. Genotypes are available for 511 such families from AGRE (www.agre.org) and 784 families from the FHS collection (http://www.ncbi.nlm.nih.gov/sites/entrez?db=gap, FHS SHARe collection). The AGRE samples consist of 2,883 individuals genotyped at 399,147 markers on the Affymetrix 5.0 SNP Chip platform. We excluded ∼3,150 markers from analyses due to deviation from Hardy-Weinberg equilibrium (P<10^−7^) or Mendelian errors. The FHS includes genotypes at 500,568 markers from the Affymetrix 5.0 SNP Chip for 9,237 individuals. We excluded ∼22,000 markers from analyses due to deviation from Hardy-Weinberg Equilibrium (P<10^−7^) or Mendelian errors.

### Determining recombination phenotype

We identified recombination events for maternal and paternal sides separately in two-generation families with at least two children using an approach similar to that of Coop et al. [3]. By looking at informative markers (defined as SNPs where one parent is homozygous and the other parent is heterozygous), we determined the number of alleles shared identical-by-descent between a “reference” child and each other child. For example, assume the father has genotype AA and the mother has genotype TA at a maternal informative marker. If two of their children have genotype TA or AA at this marker, we know they both inherited the same maternal allele. If one child is TA and the other is AA, they inherited different maternal alleles. A switch from the “same maternal allele” state to the “different maternal allele” state in the sibling pair as we move along the chromosome indicates that there was a maternal recombination (Figure 1). In a family with only two children, we cannot determine in which child the recombination occurred, so the parental recombination phenotype can only be scored as an average for the two children. In a family with three or more children we can assign each recombination to a particular child. If recombination is observed between the reference child and only one sibling, that recombination can be inferred to have occurred in the sibling. But if it is observed when the reference child is paired with the majority of siblings, it can be inferred to have occurred in the reference child. Regardless of the number of children, we scored the recombination phenotype as an average per meiosis for each parent for the purposes of GWAS and most other analyses.

To minimize spurious recombinations caused by genotyping errors, we required that each recombination event be supported by 5 or more consecutive informative markers.

The PERL module that we used for determining recombination phenotype is available for download at http://genomics.med.upenn.edu/recombination.

### Finding recombination jungles

To identify the location and size of recombination jungles in the AGRE and FHS samples, we sorted and plotted all recombination events by base pair position (Figure S1). To model these data, we used MATLAB to fit weighted piece-wise polynomial curves. To account for different sized intervals, the inverse size of each interval was used as a weight, then, a smoothing parameter (p = 0.1) was used for curve fitting. We then calculated the derivative function for the curves and used that as the relative frequency of recombination events along the chromosome. Regions with frequencies that are two standard deviations or more above the average value of the derivative function were identified as recombination jungles. The widths of jungles are defined as the regions around the peaks that are one standard deviation unit above the average recombination activity.

### Genome-wide association studies (GWAS)

To identify the DNA variants that influence individual variation in recombination phenotype, we carried out genome-wide association analysis. Since the distributions of female and male recombination phenotypes are different, for all the analyses, we studied female and male recombination phenotypes separately. All association tests were performed using the PLINK software package [21]. Recombination phenotypes were used as quantitative traits in an additive genetic model. All markers exhibiting Mendelian errors, deviation from HWE, and/or having a minor allele frequency less than 0.05 were excluded. Some SNP genotypes that were available in the AGRE data were not available in the FHS sample. For the GWAS analyses, these genotypes were inferred using the program, MACH [22]. We confirmed the significance of SNPs in Table 1 by permutations (with 100,000 and 1,000,000 replicates).

### Gene expression analysis

Mouse male meiotic cells were purified by fluorescence activated cell sorting (FACS) as previously described [19],[20]. Cells from the studied fractions encompassing the entire meiosis I and II include spermatogonia, leptotene/zygotene, pachytene, diplotene and secondary spermatocytes/round spermatids. In addition, the entire meiotic population was purified and used as the reference (calibrator) sample for the quantitative PCR. Total RNA for all six samples were extracted using the RNAqueous micro kit (Ambion) and first-strand synthesis was performed using the SuperScript III reverse transcriptase kit following suppliers' instructions (Invitrogen) from 15 ng of total RNA for each sample. The primers used for analysis are: Rnf212/F GAA AGC CTG AGA TGT CAG CAG, Rnf212/R GGC TGG CTA CAG AGC GTA GAT, Nub1/F GTT ACA GGA TGC AGA CCC TGA, Nub1/R CAT CTG TCG AGG CAC TAG AGG, Ugcg/F GAC AGA GAA AGT GGG GTT GGT, Ugcg/R CTC CTG CCT GAT CTA GCA CAT, mActinb/F ATA TCG CTG CGC TGG TCG TC, and mActinb/R AGG ATG GCG TGA GGG AGA GC (F: forward, R: reverse). Primer pairs were chosen to amplify across distant exons in order to avoid false positive amplification from contaminating genomic DNA. Quantitative PCR was performed using SYBR green mix (Quanta Biosciences) with ROX as a reference dye (Invitrogen) using Realplex Mastercycler 4S (Eppendorf) following the supplier's protocol. Melting curve analysis confirmed the simple nature of the amplified product for each gene. Relative expression (RE) was calculated following the ΔΔCt methodology with RE = 2^−ΔΔCt^ with ΔΔCt = ΔCt_Sample_−ΔCt_Reference_, and ΔCt_Sample or Reference_ = Ct_Gene_−Ct_b-actin_.

## Supporting Information

Figure S1 Recombination events and jungles on chromosome 22. Maternal (A) and paternal (C) recombination events are represented by horizontal black lines across chromosome 22. These lines “stack” upon each other in regions of high recombination activity. Derivatives resulting from curves fitted to the recombination events are shown for maternal (B) and paternal (D) data. Recombination jungles (gray) are identified at peaks in the derivative functions which correspond to regions with high recombination activity.
(4.94 MB TIF)

Table S1 Recombination events by chromosome.
(0.01 MB PDF)
