# Supplementary figures and images for: Genetic Analysis of Variation in Human Meiotic Recombination
Source: PLoS Genet. 2009 Sep 18;5(9):e1000648. doi: 10.1371/journal.pgen.1000648 (PMC2730532; doi:10.1371/journal.pgen.1000648)

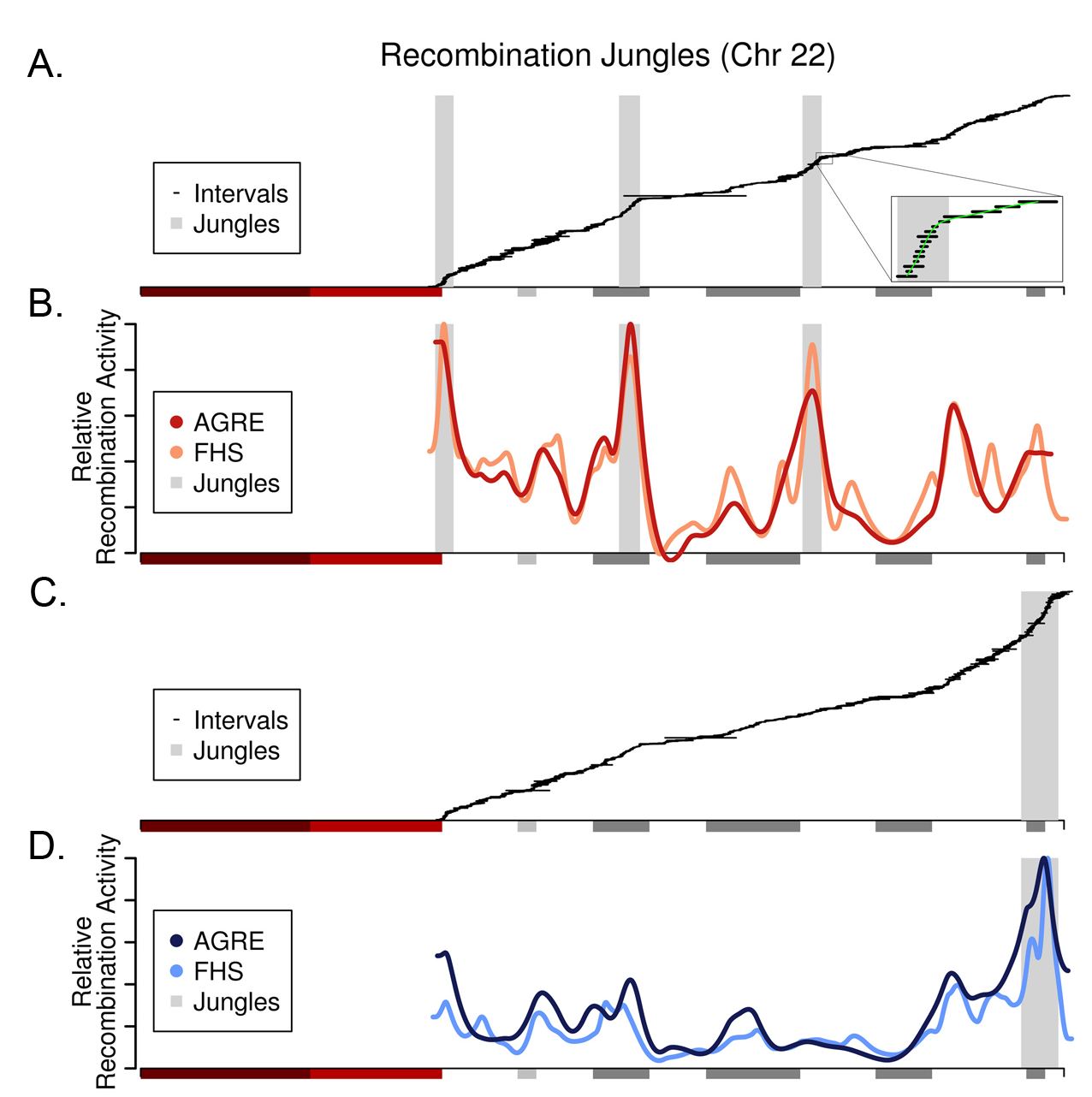

Supplement: Figure S1 — Recombination events and jungles on chromosome 22. Maternal (A) and paternal (C) recombination events are represented by horizontal black lines across chromosome 22. These lines “stack” upon each other in regions of high recombination activity. Derivatives resulting from curves fitted to the recombination events are shown for maternal (B) and paternal (D) data. Recombination jungles (gray) are identified at peaks in the derivative functions which correspond to regions with high recombination activity. (4.94 MB TIF) [file pgen.1000648.s001.tif]
