# Supplementary material for: Genetic Analysis of Variation in Human Meiotic Recombination
Source: PLoS Genet. 2009 Sep 18;5(9):e1000648. doi: 10.1371/journal.pgen.1000648 (PMC2730532; doi:10.1371/journal.pgen.1000648)
Supplement: Table S1 — Recombination events by chromosome. (0.01 MB PDF) [file pgen.1000648.s002.pdf]

Supplementary Table 1 Recombination events by chromosome

	Maternal				Paternal			
	AGRE		FHS		AGRE		FHS	
CHR	Recombs	%	Recombs	%	Recombs	%	Recombs	%
1	3801	7.99	7258	8.04	2269	7.42	4266	7.48
2	3662	7.70	6841	7.58	2241	7.33	4004	7.02
3	3121	6.56	5824	6.45	1869	6.11	3509	6.15
4	2974	6.25	5666	6.28	1675	5.48	3109	5.45
5	2859	6.01	5329	5.90	1643	5.37	3077	5.39
6	2682	5.64	5080	5.63	1558	5.10	2994	5.25
7	2541	5.34	4788	5.30	1508	4.93	2980	5.22
8	2470	5.19	4598	5.09	1498	4.90	2590	4.54
9	2164	4.55	4166	4.62	1431	4.68	2693	4.72
10	2395	5.04	4690	5.20	1493	4.88	2932	5.14
11	2157	4.54	4135	4.58	1451	4.75	2652	4.65
12	2230	4.69	4284	4.75	1537	5.03	2782	4.88
13	1753	3.69	3262	3.61	1158	3.79	2093	3.67
14	1606	3.38	3033	3.36	1156	3.78	2087	3.66
15	1566	3.29	3118	3.45	1142	3.73	2218	3.89
16	1768	3.72	3305	3.66	1223	4.00	2257	3.96
17	1753	3.69	3379	3.74	1204	3.94	2266	3.97
18	1534	3.23	3009	3.33	1089	3.56	2052	3.60
19	1360	2.86	2495	2.76	1021	3.34	1940	3.40
20	1420	2.99	2591	2.87	1074	3.51	2077	3.64
21	849	1.79	1631	1.81	591	1.93	1134	1.99
22	884	1.86	1782	1.97	747	2.44	1342	2.35
Total	47549	100	90264	100	30578	100	57054	100
